# Book Reviews

**Published:** 1822-12

**Authors:** 


### CRITICAL ANALYSIS

OF

### ENGLISH AND FOREIGN LITERATURE

RELATIVE TO THE VARIOUS BRANCHES OF

### Medical Science.

Quæ laudanda forent, et quæ culpanda, vicissim

Illa, prius, cretâ; mox hæc, carbone, *notamus*.—PERSIUS.

### DIVISION I.

### ENGLISH.

ART. I.—*Medical Report, containing an Inquiry into the Causes and Character of the Diseases of the Lower Orders in Dublin.* By T. C. SPEER, M.D. M.R.I.A. late Physician to the Dublin General Dispensary. [Dublin Hospital Reports, Vol. III.]

THIS paper is one on statistical medicine, and intended to illustrate the causes of the various diseases which prevail among the lower orders of society in Dublin; an undertaking, in the furtherance of which Dr. SPEER's situation as physician to an extensive dispensary gave him peculiar advantages. Investigations of this kind, however, must necessarily be at all times laborious, and often attended with extreme difficulty, from the complicated nature of the causes which frequently co-operate in the production of a joint effect. In hospitals, we know only the symptoms of the patient as they exist at the period of admission; to ascertain their origin is often impossible; and no opportunity is afforded of connecting certain diseases with their corresponding localities, or observing the habits of life or peculiarities of diet on which they depend. Besides, some diseases, and those of the most fatal tendency, are entirely excluded from our hospitals, so that the return of admissions can never be admitted as corresponding exactly to the prevailing diseases. In this view dispensaries, regarded as medical schools, possess considerable advantages over hospitals: disease, unaltered by change of atmosphere and regulation of diet, is seen in its worst and most varied forms. Hospitals (if we may be allowed the comparison,) are like cabinets,—they present select specimens of disease, culled and arranged: dispensaries show diseases, whether epidemic or sporadic, in their natural localities,—under such circumstances, in short, as they are met with by every medical man, when he ceases to pursue physic merely as a study, and comes to practise it as a profession. Dispensary practice (to use the words of the author before us,) creates promptitude: it affords a wide range of insight into local peculiarities; it opens an immense and diversified page, not only in the book of medicine, but of mankind; it breaks away the fancies of the closet and the bondage of the schools, and gives confidence and courage at the bedside of a patient.

Dr. Speer regards "*contagion*, taken in the wide and general sense of the term," as the "grand connecting and assimilating principle of city disease." Before we subscribe to this doctrine, it would be necessary to know more distinctly what the author understands by this "wide and general sense:" if he means that disease in great towns is chiefly propagated by direct contagion from one individual to another, then we are decidedly of a different opinion. But we entirely agree with him if he alludes to that vitiated state of atmosphere produced by crowding human beings together, particularly when labouring under disease. To remove or lessen this contamination by every possible means, is the most direct and efficient method of preserving the public health, both from the effects of individual contagion, and the more general and destructive influence of epidemics.

A general uniformity prevails in the diseases of great towns; but, notwithstanding this resemblance in the outline, much difference will be found in the detail: varieties, indeed, are found in different situations of the same town, or even in the same localities, depending on the trade, habits, character, &c. of the individuals; a position which we shall have occasion to illustrate more particularly in the sequel. The causes which particularly influence the modifications of disease in Dublin, according to the division of our author, are Climate, Poverty, Population, and National Character: these we shall take in succession.

*Climate.*—It would be a work of supererogation to describe minutely the climate of Dublin: suffice it to say, the characteristic features are great humidity and extreme variability of temperature: yet, compared with London, it is freer from extremes of heat or cold, the thermometrical range being somewhat more limited; the summers are not so oppressive, the winters are milder, and the seasons, in general, more blended into each other. Certain vicissitudes of temperature, prevailing at every season, are always liable to cause inflammatory affections; and, in this part of the history, we can perceive no difference between the climate of Dublin and other places, except as it may be more variable, and act upon subjects less capable of resisting its influence. How far the humidity of the atmosphere is concerned in the production of disease is made a question, which the author is rather disposed to answer in the negative. Dr. Rutty, who paid much attention to the climate of Dublin, thought moist seasons the most healthy; and Dr. Franklin, and Dr. Percival of Manchester, have advanced similar opinions. To this we would add, that last winter, which was so remarkable for the immense quantity of rain, was likewise remarkably free from severe forms of disease.* But the question naturally suggests itself, whether this arose from the presence of moisture or the absence of cold? We suspect it was from the latter cause. Spring, we are informed, is the season most liable to variations of temperature in Dublin, giving rise to affections of the lungs, pleura, and air-passages: "in London this class of diseases seems most abundant in winter." In this assertion we do not agree with the author: in winter we may have more acute attacks of pleuritis, perhaps, but it is in the spring we see the greatest number of pulmonary affections, because that season is here, as in Dublin, the most remarkable for its mutability.

Notwithstanding the unfavourable nature of the climate, the usual forms of scrofula are said to be much less frequent than in this country, and tubercular phthisis "infinitely more rare." There are no peculiarities described with regard to the other seasons, sufficiently striking to be worthy of notice.

*Poverty*.—Among the many avenues opened to disease by poverty, diet is regarded as claiming the first attention.

"The quantity and quality of this are alike poor; the principal articles seem to be potatoes, salt fish, and tea. Potatoes are the grand nutriment principle and support of existence, and without this invaluable vegetable hundreds must daily drop into the grave. Always a favourite, and always easily obtained, it forms the great barrier to the ravages of hunger, and indeed constitutes almost the only one. Next to the potatoe, salt food seems the favourite article, particularly salt fish. Flesh meat can seldom be procured, and of this the salt kind is preferred, and particularly bacon. Of fish, herrings are the favourite species. This attachment to salt food probably arises from two causes: first, its greater cheapness, and, secondly, its stimulating and sapid quality. It is impossible not to notice the preference which the lower orders here, compared with those of England, have to *stimulating* or flavorous, rather than *nourishing* food, and indeed their carelessness about the latter, except potatoes. This may be partly explained from their national character, as will hereafter be mentioned. In the diet of the lower orders in England, nourishment is the grand object, and the rules of their diet are conducted with a system and an arrangement completely unknown here. With us, what excites is the chief consideration; and, as to regularity in meals, the poor are very indifferent about it. With the former, the *stomach* is the presiding organ; it seems to hold dominion over all the others, and to be a complete tyrant.

---

* See Report of the Diseases of London.—*London Med. and Phys. Journal*, April 1822.

With us, the *nerves* appear to hold this place, and to these a great deal is sacrificed. It is impossible not to notice, with the lower orders in England, when ill, that their great and first complaint is 'they can't eat;' here the general complaint is, that 'they have a fluttering or oppression about the heart.' Although in the large towns and cities there, as in Scotland, this distinction may not be strong, and although the proportion of stimulating to nutritious food and drink is much greater from various causes, yet the latter is always a paramount object of attention with them; they conceive that eating alone produces strength and health, and they find the same degree of pleasure in it that the lower orders here do in drinking. With us nutrition is slighted, except in potatoes. Bread, cheese, and oatmeal give way to this vegetable, in like manner as porter and ale give way to whiskey. With the lower orders in France, bread constitutes the grand nutrient principle, and of this immense quantities are consumed. With those in Scotland, oatmeal and potatoes are the chief articles of food, and in both countries great regard is always paid to nourishment. Notwithstanding, however, this carelessness among our lower orders, they are capable of labours and fatigues equal, if not superior, to the others, and nature appears to have eminently gifted them with the hardiest and most vigorous constitutions. We know that in London the hardest species of labour are often confined to the lower orders of the Irish, and in our own streets we every day see surprising loads and burdens carried by women."

We agree in the general accuracy of this account, but the parallelism is, nevertheless, in some points incorrect. It is true that the inhabitants of this country may be more particular about the quality of their food,—the natural result of having some choice; yet the assertion that they regard "eating alone" as the source of health and strength, is a mistake. In London particularly, the quantity of porter and spirits consumed by men of laborious trades is immense; and we have frequently heard its use justified on the plea of necessity. And when Dr. Speer argues that the Irish in London perform the most fatiguing species of labour, notwithstanding this carelessness of diet, he forgets that his countrymen, when they migrate, acquire, a taste for better living, with wonderful facility. In short, we do not think, as Dr. Speer seems to do, that Irishmen, fed on potatoes and whiskey, are stronger than Englishmen who live on roast beef and porter.

From the strain of panegyric in which the author speaks of tea, we should be inclined to suspect that he was almost as partial to it as Dr. Samuel Johnson. "Unlike its more dangerous rival, whiskey, its draughts, though impoverishing, are not delirious; if it drowns sorrow, it does not drown sense; if it gilds the gloom of poverty, is not the delusion a blessing? It seems, indeed, the general panacea, always affording comfort, calmness, and consolation; constituting not only the leading article of breakfast and supper, but often of dinner; and over its placid inspirations their happiest hours seem to be passed." The author has put what seems meant to be assertion in the indirect form of an interrogatory, which we must take the liberty of answering otherwise than he intended. *If* it gilds the gloom of poverty, it must be a blessing: but, on the other hand, we find, a few pages further on, that it gives rise to "low nervous fevers, great debility, tremors, palpitations, vertigo," and sundry other mischiefs; and we ourselves think we can trace many of the dyspeptic diseases, which abound among the lower orders in London, to the passion for tea,—or the various combinations of herbs sold under that name.

Whiskey comes next under consideration, although we are told, we know not exactly why, that it might rather be placed under the head of national character or climate. Spirits of some kind are used in every climate, and all uncultivated nations show a predilection for them, as may be found by consulting the writings of travellers and navigators; and, with regard to national character, the only people who can rival the Irish in the consumption of whiskey are the Scotch highlanders, than whom no people bear less affinity in temper or disposition. Dr. Speer is evidently a friend to whiskey: that the *use* of it is attended with great advantages, he thinks unquestionable; but that its *abuse* has thrown these advantages into discredit: and again, "the bad effects, however, resulting from this fluid with us are not, I think, to be ascribed to the quantity consumed." He is of opinion that the inhabitants of the manufacturing towns in this country indulge in more copious potations, but that they do not suffer so much, because they do not "take it on empty stomachs;" by which means they prevent it from affecting the coats of the stomach, or impressing its nerves so directly. The principal consumption of whiskey, however, in this island is in the highlands of Scotland, where it is illicitly distilled of great strength, and a glass of which it is the invariable custom, in many places, to take before breakfast, as well as during the day, whenever any tolerable pretext can be found. We are aware that Dr. Mac Culloch has described dyspepsia as very frequent in the Highlands; yet it is extraordinary how little apparent mischief sometimes results from this practice. The late very distinguished Dr. Gordon, of Edinburgh, had, before his death, collected a great number of instances in which individuals who habitually drank whiskey to excess, enjoyed good health, and attained advanced age. Notwithstanding that our author thinks the inhabitants of some parts of England and Scotland drink more, still he allows the consumption of whiskey in Dublin to be "immense," and grievously laments the toleration of so many public houses. This evil, we fear, is equally prevalent in all our great towns, and must continue, being one which, "touched by the Midas finger of the state, bleeds gold" —for the revenue. The diseases which Dr. Speer has observed to result from the abuse of whiskey, have their seat in the stomach and liver, but especially in the latter. These remarks differ in nothing from the effects generally attributed to spirituous liquors.

Poverty, besides giving rise to unwholesome diet, is the indirect source of many other predispositions to disease, particularly from bad accommodation and clothing. Cutaneous affections, with the whole class of the cacheciæ, are consequently numerous.

*Population.*—This seems not to depend so much on poverty, and its consequent evils, as might be supposed; for, notwithstanding the details of misery which precede, yet, according to our author, the propagation of the species flourishes surprisingly in Dublin. In 1798, the inhabitants of Dublin were estimated at 170,805. Dr. Speer calculates them at 250,000; the census of 1821, at 238,201; being a difference, in the estimates, of 11,799. The causes of this increase in the population, and the apparent decrease in the means of its subsistence, are supposed to be, directly or indirectly, nearly the same,—viz. the return of peace, the nearly extinct state of manufactures, the decline of trade and wages, and the want of employment for the poor; added to these, early and improvident marriages. Now, that this last may, and frequently does, occasion the getting of children, is very evident, and easy of comprehension; but the former causes assigned appear to us rather paradoxical. Indeed, the only way in which we can suppose the want of employment, &c. to operate, is that the people set about getting children because they have nothing else to do.

To whatever causes it is to be attributed, no doubt can exist as to the fact of the number of inhabitants having increased generally, and recent calamitous events have shown the miseries which are liable to result when the produce of a country is not in proportion to its population. These, however, are but temporary, though dreadful, visitations, and carry their own cure in the devastation they produce: our more immediate business is with the effects of over-crowding in large cities. The first and most striking of these is the rapid progress and fatality of epidemic diseases, the dwellings of the poor becoming hot-beds of infection, which keep up the disease long after its dependence on the state of the general atmosphere has ceased. In London, by far the most crowded and filthy districts are those inhabited by the Irish labourers: some of these exist in the very centre of the town, in the neighbourhood of frequented streets, and are passed every day by thousands who never suspect their existence. Such, for instance, is the whole range included between

Russell-street and Broad-street, St. Giles's; a part of the town which few visit from inclination, and none without astonishment that, amid the great improvements which have recently been made, such a focus of disease should be permitted to exist. The streets are the receptacles of every variety of filth; the houses generally let out like barracks to labourers, having from three to six beds in each room, occupied by two or more tenants; having no air but what has constituted "the most sweet breath" of its inhabitants, times without number. Nay, we once had occasion to attend a patient labouring under typhus in this district, in a cellar having neither window, chimney, nor any other opening, except a small door by which it communicated with the external world. Some may think the picture too deeply coloured; but those whose professional duties have called them into the various parts inhabited by the poorest of this metropolis, will not fail to acknowledge its fidelity. The same scenes may likewise be found in the capital of the sister kingdom.

*National Character.*—The peculiarities of this, so far as they may be supposed to influence disease, chiefly consist in the predominance of impetuous feeling, lively imagination, warm affections, and a tendency to hope for the best. The first renders them subject, in an eminent degree, to those affections called nervous; and we have frequently had occasion to remark that the Irish refer their ailments to the heart much more frequently than the English or Scotch. The last makes them bear up against misfortune; and we must do them the justice to say that we never have to listen, in our professional capacity, to a grumbling detail of gloomy forebodings, which in other cases is often added to the history of the complaint.

Although the Irish are capable of great exertions, yet indolence is a prominent feature in their national character, and a want of cleanliness follows as a natural consequence. In this respect they bear a striking resemblance to the Scotch, and in both countries cutaneous diseases are the result. But, besides the carelessness in their persons, it appears, from the account before us, that the same indolence gives rise to the most culpable neglect in removing from their dwellings and streets those excrementitious and other putrescent matters, the effluvia of which must necessarily prove the source of epidemics, and facilitate the operation of contagion.

Art. II.—*Fatal Consequences resulting from slight Wounds received in Dissection.* By A. Colles, m.d. &c. &c. Professor of Anatomy and Surgery in the Royal College of Surgeons in Ireland. [Dublin Hospital Reports, Vol. III.]

This paper contains the detail of three interesting cases, with reflections arising from them, which we recommend to the attention of gentlemen entering upon courses of practical anatomy.

Mr. W. Hutchinson, of rather delicate constitution, received a slight scratch on the outer side of the first phalanx of the right thumb, with the knife used in opening the body of a man who had died of cynanche laryngea. The wound did not exceed one-sixth of an inch in length, and was otherwise so slight that he scarcely noticed it at the time. The cellular membrane in the body examined contained, on the external surface of the larynx and trachea, "that amber-coloured fluid, resembling melted jelly, which is so often met with in such as have been carried off by this disease." In the evening, Mr. H. was drowsy, and retired earlier than usual in consequence. Next morning, he was affected with head-ach, sickness at stomach, and very acute pain in the right shoulder and axilla; which symptoms increasing, he took an emetic, and followed it up with a purgative dose. On the third day, the pain had increased very much, and was confined to the shoulder-joint, in the neighbourhood of which there was some swelling, but without any discoloration of the integuments. The wound was quite free from inflammation, a small vesicle having appeared, which was flattened, and about half filled with a milky effusion. No inflamed lymphatics could be discovered, nor any enlarged glands. He had much fever, and great dejection of spirits, The usual febrile remedies were employed, fomentations were applied to the parts, and leeches to the axilla, without effect; nor did large doses of opium alleviate the pain. In this state he continued for several days, when at length some remission of the pain took place, but unaccompanied by corresponding diminution of the fever.

In the course of a few days more, he complained of pain along the same side of the thorax, and an erysipelatous redness was perceived, running from the axilla nearly half way to the ilium: this redness soon extended downwards to the trochanter, and forwards to the groin; the integuments having a doughy feel, and receiving the impression of the finger. The surface presented, in some places, the appearance of vesicles; but these, on more minute examination, proved to be little solid elevations. The patient's strength had now become much exhausted, and was supported only by large quantities of wine. On the 15th of December, an incision was made over the fourth and fifth ribs, in hopes of giving vent to some effusion, but without this effect resulting; nor did the operation at all affect either the local inflammation or general symptoms. The pain in the shoulder had previously ceased, and at the end of the third week that of the side likewise yielded; but at this time he was attacked with pain (which soon became very violent,) along the inner edge of the biceps muscle; the arm became swelled and inflamed, some induration extending even to the pectoralis and latissimus dorsi. Fomentations and poultices were applied, and, on the 1st of January, an incision made at the inner side of the arm, where some degree of fluctuation was perceptible. This gave vent to a small quantity of matter. Another abscess formed on the side; after which he rapidly got well, although the arm did not entirely recover for some weeks.

Mr. Dease, late professor of anatomy and surgery to the Royal College of Surgeons in Ireland, demonstrated the cervical nerves and brachial plexus in a woman, about forty years of age, who had died about eight-and-forty hours before, of a chronic disease of the lungs. The subject had been dissected for him. Next morning (February 14, 1819,) he awoke early with rigors and sickness; he vomited a quantity of fish, on which he had dined the day before, and afterwards bile. At half-past eleven, Dr. Colles saw him, when he regarded the case as a severe attack of the fever at that time epidemic, attended with unusual derangement of the digestive organs. He had complained, all the morning, of violent pain in the left shoulder, for which he earnestly desired to be bled; and his request was complied with at three o'clock P.M. Twenty ounces were taken from the arm by a large orifice; the blood showed no inflammatory coat. No decided relief was afforded, even at the time, and by nine o'clock in the evening the pain and fever were as great as before. A slight degree of fulness was now discovered above the clavicle, which proved exquisitely painful on the slightest pressure.

*Monday*, 15*th*.—He had spent a wretched night, from pain in the shoulder; and, when Dr. Colles visited him, "had the entire joint covered with leeches, to the amount, perhaps, of one hundred!"

From this day he appears to have been attended by Dr. Sheridan, Dr. Brookes, Mr. Richards, and the author. A draught, (what?) purgative pills, fomentations, opiate liniments, and enemata, were ordered. The draught was rejected, but the bowels were opened by the pills.

At nine o'clock next evening, he mentioned that he felt some uneasiness about the side; and, upon examination, a colourless swelling was observed, a little behind and below the posterior border of the axilla. This naturally led to the inspection of the hand, when a slight scratch, not one-fourth of an inch in length, was discovered on the thumb, and a small vesicle had formed on its site, half filled with a white effusion. This had made so little impression, that he denied at first having received any wound whatever. The pain and tenderness on pressure above the clavicle had diminished, but continued in the side, at the part where it was swollen; and the restlessness and depression were observed to suffer an exacerbation every evening about six o'clock. He took purgatives, opiates, diaphoretics, and ammonia, at different times, and seemed to experience relief up to Friday the 19th; when, after a very restless night, with some delirium, Dr. Colles discovered a vesicle on the fore-arm, about two inches below the incision made in bleeding him: the orifice of the vein was "inflamed in the ordinary way." Next day, his manner was quick, and his pulse continued to get smaller. The tumefaction now existed from a little below the arm-pit to the hip; it was covered with the same little hard elevations, resembling vesicles, which were mentioned in the case of Mr. Hutchinson; a slight degree of erysipelatous redness now occupied a small portion of the centre of the swelling, and by nine in the evening had spread considerably; the swelling, at the same time, extending towards the back. Some fulness of the abdomen seems to have prevailed throughout, but the bowels were kept free.

Saturday the 20th, he was very delirious, and insisted on being moved into another bed, where he lay nearly three hours without any clothes. Next day, a poultice was applied to his side, (the inflammation continuing to extend,) and cordials exhibited. A swelling was now observed in the right fore-arm, beginning about an inch and a half below the orifice in the vein, and extending about "a hand's breadth" over the flexor muscles; but no change had taken place in the vesication already mentioned. This tumor was punctured at five o'clock P.M. and about a teaspoonful of serous fluid escaped, but without the slightest reduction of the swelling.

"*Sunday evening, nine o'clock.*—Pulse in right wrist not to be felt; heat of limbs not reduced; breathing quick and laboured. He passed urine at five o'clock this evening; and *at ten o'clock he died.*"

On this side of the Irish Channel, we prefer dating the report which contains an account of the death of the patient after, rather than before, the event has occurred: but this *en passant.*

The morning after his death, two or three vesicles were observed on the back, and the raised hard spots continued unaltered.

Mr. Egan dissected, on the same day (February 13th), part of the subject which Mr. Dease had used for his demonstration. Next day, he was attacked with rigor. On the 16th, the metacarpal part of the thumb was inflamed, with pain extending up the fore-arm: he was hoarse, and had considerable fever. By the 28th, the symptoms had greatly increased, with severe pulmonary "affection and distress." An abscess, which had formed in the axilla, was now opened, from which purulent matter, of unusually thick consistence, was discharged; the cavity extending from the pectoralis to the latissimus dorsi. From this time he gradually recovered.

These cases are of great interest, and we have given them as distinctly as the abbreviations which our limits demand would permit us: indeed, we have dwelt upon them more than we might otherwise have done, because, although serious, and even fatal, consequences have frequently occurred from wounds received in dissection, yet we have not been able to find any description corresponding to that before us, either on reference to books, or in conversing with our anatomical friends upon the subject. The author also seems to regard the train of symptoms as new in pathological record.

Mr. Dease had been in the habit of practising dissection for twenty years, so that no length of time, nor extent of habit, can be regarded as protecting the constitution from the invasion of this disease. The subject, it will be observed, was recent, and, except a thick brown fluid contained in the pericardium, there is no evidence of any thing remarkable about it: this shows the evil to be unconnected with putrefaction. Dr. Colles alludes to this circumstance, and to the general opinion, even among medical men, that the danger arising from wounds in dissection is in direct proportion to the degree of putrefaction; but, on the contrary, according to his observation, unpleasant consequences have been so rare where the subject was far advanced in putrefaction, as to induce him to think that it rather gives protection to the anatomist. There is, in our opinion, much truth in these remarks: were serious mischief to result often from scratches received in dissecting putrid subjects, the anatomical schools in this metropolis would be deserted. For our own part, we have dissected, and seen others dissect, bodies in every stage and degree of putrescence, with perfect impunity; but have repeatedly suffered severe pains in the hands shooting up the forearm from opening bodies soon after death; and we think the sooner after death such examination takes place, the greater liability there is to this occurrence. This is a remark which we have often mentioned, on these occasions, to the pupils at the Westminster General Dispensary. The observation, however, is not new, as we remember to have heard it mentioned, in his Surgical Lectures, by our learned and ingenious preceptor, Dr. THOMPSON, of Edinburgh.

In order to prevent these inconveniences, Dr. Colles recommends immediately dipping the finger into oil of turpentine. This is a point, however, on which some difference of opinion seems to exist; M. PATISSIER and M. LAENNEC having both, in recent works, advanced the opinion that washing carefully with cold water is more to be trusted than burning the parts with caustic. In order to determine a question of this kind, it is necessary to form a distinct idea of the object in view by any application made use of. This, we conceive, to be simply to prevent the poison from being absorbed, and it may be effected in two ways,—either by removing the poison altogether, or by rendering the vessels incapable of taking it up. The plan of ablution can only operate in the former manner, and, where it is complete, must obviously render the second unnecessary; but then, unfortunately, the poison is not an object of sense, and therefore we have no means of ascertaining its removal. Is it not more prudent, then, to act as if it still remained, and, after careful ablution, to destroy the wounded surface with caustic? Such is the opinion of M. CHAMBON, who has paid much attention to the subject, having himself suffered twice very severely from this unpleasant accident. Various kinds of caustic application may be used, but those in the liquid form are preferable, from pervading with facility the whole of the cut or puncture; and, perhaps, there is none better than the solution of caustic alkali, so much praised by MEDERER DE WUTHWEHR: not, however, from the vulgar idea of its neutralizing the poison, of which no evidence exists, but, as we have mentioned above, on the principle of destroying the surface to which the poison is applied, and thus rendering its absorption impossible.

### DIVISION II.

### FOREIGN.

Art. III.—*De l'Usage du Moxa, (Recueil de Mémoires de Chirurgie,* par M. le Baron Larrey.) Paris, 1821.

Art. IV.—*On the Use of the Moxa as a Therapeutical Agent.* By Baron D. J. Larrey, &c. &c. *Translated from the French, with Notes, and an Introduction containing a History of the Substance.* By Robley Dunglison, Fellow of the Royal College of Surgeons, &c. 8vo. pp. 148. T. and G. Underwood, London, 1822.

There is no greater distinction between ancient and modern surgery, than the almost total abandonment of the use of the actual cautery in our times; no remedy having been more extensively and universally employed, in all its different forms and modifications by our forefathers. In this respect the learned and unlearned nations of the ancient world were agreed; Greeks and barbarians, all equally called in the aid of fire, in one shape or other; and there seem to have been few parts of the body exempt from its operation, and scarcely any disease in which its efficacy was not acknowledged.

Those who are fond of beginning from the beginning, may, if they please, found their practice upon the authority of Hippocrates, or even that of the centaur Chiron; and we cannot deny to the Father of Physic the merit, or the indiscretion, of having urged the medical use of fire in the strongest and most forcible language; a language which, indeed, from its conciseness, leaves ample room for discussion and explanation, and which a modern surgeon may fairly be permitted to object to; since, in these days, we do not usually endeavour to destroy, either with the knife or cautery, all those diseases for which our assistance is demanded, and we should think it rather an abuse of language to call such a compendious mode of treatment a cure. Operative surgery is certainly a great triumph of art; but, after all, it is a triumph for which, as philosophers, we may be allowed to blush, since, in almost all the instances in which we call the knife to our assistance, we, by so doing, acknowledge our inability to *cure* the disease, which we are therefore obliged to remove, together with that portion of the living body which is the seat of it.

The use of fire in medicine and surgery admits of two leading distinctions: 1st, the sudden application of substances heated to such a degree as to cause an immediate destruction of the parts to which they are applied; and, 2dly, the more tedious combustion of inflammable substances applied to the skin, and in which latter class the moxa must be arranged. The memoir of Baron LARREY upon the employment of this latter remedy, which Mr. DUNGLISON has presented to us in an English dress, is almost exclusively confined to this species of cautery; but, before we proceed to analyse this work, we must be permitted to say a few words generally upon the use of the actual cautery, strictly so called, and to inquire whether English surgeons are justified in having so entirely laid aside this formidable, but long established, remedy; a question, one would conceive, not very difficult to decide, since the application is usually made for the cure of some obvious and apparent disease, and in which the benefit derived is clearly traceable to the means employed; unlike the effects of internal remedies in internal diseases, where the cure, although following the remedy, is not always in consequence of its use. The rapid succession of favourite medicines,—their short-lived fame, and long oblivion, sufficiently illustrate the truth of this position.

It is not surprising that cauterization should have been resorted to in the infancy of our art, and by nations in a state of barbarism: the potency of the action of fire, and the impossibility of curing many diseases, in those days of ignorance, without a total destruction of the diseased parts, must have been urgent motives for the adoption of this severe treatment. The Greeks transmitted their knowledge of this practice to the Arabians, who took it up with all the fervour belonging to the character of that enthusiastic people, and not only applied it more extensively than their masters, but refined upon the practice to such a degree, as to ascribe different powers and merits, not only to the different shapes of their cauterizing instruments, but also to the very materials of which they were composed; so that gold and silver were frequently made use of by them in these operations, from the supposed inherent qualities of these more noble metals. Notwithstanding all these absurdities, and the natural repugnance which every one must feel to the application of red-hot substances to the skin, the use of the actual cautery was continued even down to the middle of the last century; although latterly it had certainly been applied more scientifically, and much restricted in its application: the blind deference paid to the doctrines of the ancients contributed, no doubt, greatly to preserve its reputation. In the last century, however, DIONIS in France, and SHARP in England, gave this practice the *coup de grace*. Nothing can be more positive than the manner in which Dionis decides upon the merits of the actual cautery: after having given an engraving of six different forms of these instruments, he says, "Je ne vois plus aucun chirurgien qui les mette en usage, et si je les ai fait graver ici, c'est plutôt pour vous en donner de l'horreur que pour vous conseillier de vous en user."*

The same author, in speaking of the distinctions that had been so much insisted upon between the effects of the actual and potential cauteries, expresses himself in the following manner, which is equally remarkable for the good sense and dry humour which distinguish this writer:—" Il y a quelques médecins qui ont voulu que cette distinction fût chimerique, prétendans qu'il n'y a point de cautere potentiels, et que tout cautere est une chose dont l'action est de brûler. Nous autres chirurgiens qui ne sommes pas obligés d'en savoir tant, nous en avons tousjours fait une distinction, parceque le potentiel ne brûle pas d'abord comme fait l'actuel, mais quelque tems après, en se fondant, et on nous permettra de la continuer, parceque cette distinction est tournée en habitude, et que le raisonnement contraire est si philosophique, qu'on auroit de la peine a le comprendre."

These judicious surgeons considered that all the advantages to be derived from cauterization were equally procurable by the use of caustics; and, far from attributing any particular virtue to the act of burning, they looked upon the establishment of the suppuration as the source from whence all the benefit was to be derived. The French Academy of Surgery have twice made the actual cautery the subject of a prize question; and, subsequently to the first of these periods, POUTEAU, by translating the works of PROSPER ALPINUS, brought the use of the moxa into vogue, and also warmly advocated the revival of the cautery itself. M. LOUIS and M. FAURE, about the same period, took a favourable view of the effects of this remedy; and the former, who obtained the prize assigned to the best essay upon the subject in 1755, recommends its adoption in a variety of diseases; but, as his speculations in its favour are chiefly theoretical, their authority is of the less weight. M. Faure's method of healing diseases, especially old ulcers, by fire, was somewhat different from the actual cautery, and a perusal of his paper† will convince us that his success was more to be attributed to the simplicity of his treatment, and his dispensing with the farrago of balsams, oils, and turpentines, with which surgery was then encumbered, than to the action of heat. His method consisted in applying hot coals close to the ulcerated or diseased part, and gradually bringing them as near to the surface as the patient could endure. In this manner he succeeded, in many instances, in exciting a kind of inflammatory action of the parts, which sometimes occasioned the absorption of indolent tumors, or hastened the cicatrization of languid ulcers.

* DIONIS *Cours d'Oper.* p. 837.

† *Mem. de l'Acad. Roy. de Chir.* tom. v. p. 82.

M. Peray, in renewing M. Faure's experiments, employed a flat cauterizing iron instead of the charcoal, which soon ceases to give out any heat, and consequently requires to be perpetually renewed.

It is scarcely necessary to do more than mention another mode of cauterization, which was in great vogue on the continent at no distant period: it consisted in concentrating the rays of the sun upon a diseased part, by means of a burning-glass. This produced a superficial eschar, and was said to be of wonderful efficacy in a variety of complaints. It is a remedy, unfortunately, not of every-day application in our climate, where this great cautery (the sun,) is frequently not at our service for several weeks in succession; and, besides, this method of cauterizing, as well as the practice of what was called "insolation," bears too much the character of empiricism and trick to permit us to dwell longer upon them here.

The revival of the cautery in France is, perhaps, in a great measure owing to Baron Percy, whose memoir on this subject obtained the academic prize in the year 1790. In that work, he considers the subject in all its bearings; he distinguishes the diseases to which it is applicable, and reduces the number of cauterizing instruments to five or six. Although the employment of the moxa seems to have, in very many instances, superseded the use of the hot iron in that country, still it is very frequently had recourse to for the removal of fungous excrescences, for the cure of hospital gangrene, in caries of the bones, in certain diseases about the anus, and for the destruction of the nerves of carious teeth. This is, indeed, but a meagre list, compared with the long catalogue of diseases for which the Baron has urged its employment; but the reign of moxa has just begun, and our vivacious neighbours have embraced this remedy with all their characteristic eagerness and enthusiasm.

We are reproached by continental surgeons, and more especially by the French, with ignorance of the transcendent merits of this *heroic* remedy, in all its branches; and this charge has lately been repeated by a gentleman of much talent and eminence, the Professor Maunoir, of Geneva. Now, in reply to this accusation, we have to urge the following considerations:

The operation itself of applying red-hot instruments to the living body, is so revolting to human nature, that unless it be proved to be attended with advantages not procurable by other means, that reason would be alone sufficient to exclude it from practice. That such is the fact is pretty evident, from the tricks that have been proposed by some French authors in order to cheat the patient into a belief that the iron was only to be made comfortably warm.

The pain attending the application of the actual cautery to the skin is very severe, notwithstanding all that had been urged by Pouteau and others to the contrary, and which those who have experienced an accidental burn well know; although we readily grant that neither this argument nor the former constitute any objection of weight, if counterbalanced by beneficial results not to be otherwise obtained.

The advantages arising from the tonic powers of the fire, and its local or general action, are merely hypothetical, and any thing but satisfactory to a practical surgeon; and we cannot look upon that man as well grounded in the principles of his profession who relies much upon "l'action tumultueuse du feu;" for, after all, if cautery is to do good in chronic cases, it can only be in consequence of the suppuration established, and if the benefit, on the contrary, is to arise from the sudden destruction of a diseased surface, then this tumultuous action of the fire goes for nothing. These reasons appear to decide the question against the revival of a practice coeval with barbarism, and which had gradually sunk before the advances of civilization and refinement; and, among the superior advantages which those who are fond of this remedy insist upon, we can find but one which really has a semblance of superiority, and that is the total and immediate destruction of the part cauterized, which may occasionally be desirable; although the more slow operation of the potential cauteries, and the power they possess of penetrating and insinuating themselves by their fluidity, gives them greatly the preference in the majority of instances; and we conceive that the use of fire as a remedy is not likely to be revived in this country, by any of the arguments and writings yet adduced by our neighbours in support of it.

Among the instruments which Baron Percy recommends is the cauterizing knife, or rather hatchet; and his description of his method of employing it is so similar to the plan of firing horses, as practised in this country, that we may dispense with any further account of it. We are at a loss to guess what class of people, or in what description of affections this barbarous plan is to be pursued, when we are in possession of so many milder methods of removing those diseases for the cure of which he so unmercifully sears his unfortunate patients.

There is no class of surgical maladies in which the actual cautery has been more advocated than in caries of the bones, the cylindrical ones especially; but, whoever will take the pains to read what MM. Louis, Percy, and other modern writers, have urged in favour of that method, will, we think, rise from the perusal with the conviction that the practice is uncertain; by no means free from hazard; and that, unless it be done very cautiously and temperately, it is more likely to extend the evil than to hasten exfoliation; and that, after all, what SHARP* has urged upon this very point has never yet been satisfactorily answered. We suppose that it is not necessary now to insist upon the impropriety of employing the actual cautery as a means of restraining hemorrhage. In the solitary instance of bleeding from the alveolar process after the extraction of a tooth, it may sometimes be advisable; but, wherever a ligature can be applied, no other mode of stopping a flow of blood should ever be had recourse to.

We may now dismiss the subject of the actual cautery, and turn our attention to the work which is more especially the object of our present inquiry: it is an extension of the article *Moxa*, written by Baron LARREY for the Dictionnaire des Sciences Medicales; and, in fact, although much dilated and increased by the recital of numerous cases, it contains but few observations that are not to be found under the head of Moxa and Moxibustion in the above-mentioned work. Mr. DUNGLISON, the translator of this book, has prefixed a long chapter of introductory remarks, containing a copious history of the substances employed by different nations and at different periods, to which the general name of moxa is now indiscriminately applied by continental surgeons, although that term was formerly restricted to the substance made use of by the Chinese and Japanese.

Mr. Dunglison commences his history of moxa with some preliminary remarks upon the rapid succession of new remedies, which, after having been ushered into practice with the loftiest pretensions, "in a short space of time sink into that oblivion which they frequently so justly merit," and which the attraction of novelty, and the self-love of those who delight to appear in the character of great discoverers, sufficiently accounts for. On this point of his argument our author displays a great deal of reading, and quotes authorities now quite obsolete, and examples of the existence of which few that are unacquainted with the writings of the ancient authors in medicine can be aware. We are afraid that, to the long catalogue which he adduces of the absurd prescriptions of our forefathers, a considerable addition might be made, culled from the practice of times not very remote; yet we cannot help thinking that when a remedy has, after a long continuance of favour, been gradually declining in reputation, and at length becomes extinct, that, in all probability, the arguments and facts against its employment are founded in truth; and in that predicament we have already said we conceive the actual cautery at present stands. The translator very candidly admits, that moxa appears to him to have been over-praised, and that some of the beneficial effects ascribed to it by Baron

* *Treatise on the Operations of Surgery*, p. 45.

Larrey are, in truth, too highly rated; but yet he is of opinion that sufficient evidence of its merit remains to justify a fair and full trial, which, in this country, it certainly never has had. We may here take occasion to remark, that the translator appears to us to have executed his task faithfully; the notes he has subjoined are useful, and many of them judicious; and if he has not altogether avoided some foreign idioms, yet he has upon the whole succeeded in doing what he purposed to do, in a manner very creditable to himself.

Before we begin to speak of the history of moxa, there is one circumstance which we wish to mention, because it is not alluded to either by MM. Larrey, Percy, or the translator, and that is the employment of the Chinese moxa as a styptic; yet it is quite certain that much of its reputation in the East is derived from its power of restraining hemorrhage. We have had occasion to see a large quantity of this substance, which was brought from China by the surgeon of one of our China ships, not made up in cones for the purpose of burning, but in its cottony form, and simply with the recommendation of its great virtue as a styptic. Some of this substance is now in our possession. Now, although we are decidedly inimical to the adoption of any other mode of restraining hemorrhage than that of the ligature, we can still conceive some few occasions upon which the use of a remedy really possessing the powers ascribed to moxa, may be of singular advantage; and we are assured, by a gentleman of undoubted veracity, that he has employed this substance in the case of a lady of fashion, now deceased, who suffered from an extensive cancerous ulcer in the breast, and from which, occasionally, severe and alarming hemorrhage took place: this the application of the moxa repeatedly and instantly restrained. This is one of the few cases in which such applications may be advantageously had recourse to; and the experienced surgeon will readily acknowledge that it is no less applicable to bleedings from phagedenic ulcerations in the groin or on the penis, and where it is neither prudent nor possible always to secure the bleeding vessel. We mention these circumstances because, from the respectability of the party from whom we have derived our information, we have no doubt of the facts being substantially correct; and because we are not aware of this property of the moxa being at all generally known. The reader will probably recollect that the agaric, which is used by some nations as a slow cautery and moxa, has also had great reputation as a styptic; so much so as to have formed the ground-work of many experiments, both in this country and in France, and indeed to have been relied on (very weakly, we think,) several times after amputation. Mr. Warner's cases are no doubt in every body's hands, and the volumes of the French Academy of

Surgery attest the great sensation that the novelty of the remedy produced in that country about the same period. It may, however, be objected to us, that the cottony matter of the *artemisia latifolia*, or the agaric, possesses in reality no more styptic properties than starch or flour, tinder or lint, all of which have been successfully employed in many cases of slight hemorrhage; but, if our information be correct, the fact is quite the contrary, and the moxa performs its office of styptic, not by its quantity or by its power of absorbing the blood, and thereby hastening and perfecting the formation of a coagulum, but by some specific property of procuring an instantaneous contraction of the mouths of the bleeding vessels.

We stand somewhat in apprehension of being accused of credulity, but we have given the above information as we have received it. The circumstance is both novel and curious; but we candidly admit that we have no experience ourselves of the styptic powers of moxa to offer to our readers.

We will now leave this digression, and pursue our analysis of the work before us. The authorities that Mr. Dunglison has quoted prove the general use of this slow mode of cauterization among the nations of the old and new continents, modern as well as ancient; and it appears that dried rushes, agaric, cotton, dried and rotten wood called *punk*, have all been employed for this purpose. It has already been stated that the Chinese cauteries, called moxa, are made from the dried leaves of the *artemisia latifolia*, which is gathered about June, hung up in the open air until perfectly dry, and then powdered, the *tomentum*, or down, being carefully separated from the coarser fibres. This is then made up into small cones, or folded in paper, equably and compactly put together, from which pieces are cut off with the knife, about the thickness of two quills. The Chinese and Japanese burn with these moxas both old and young, rich and poor,—none but women with child are spared; and it is as much used for the prevention as for the cure of diseases. Even those who are condemned to perpetual imprisonment are treated by being taken out of their dungeons, once in six months, in order to undergo this process. In the northern provinces of China, we are told that deep punctures are first made in the body, upon which balls of moxa are burnt. These punctures are made with needles, and the skill is to determine their number and depth. We are rather startled by the information that these *deep punctures* are not to draw blood: but this rests on the authority of the Abbé Grosier, and we fear the prop is but slender.

We need not dwell upon the names by which these operations are designated in China and Japan; nor is it a matter of great importance to settle whence the term *moxa* is derived, though it is probably of Portuguese origin. Neither need we describe the magical method of lighting the cones, nor their mode of treating the eschar: all these points we may be supposed to understand, quite as well as our brethren of Japan. It will be seen that the moxa made use of on the continent, is in imitation of the *ustio Arabica*, that form of cauterization practised by the wandering Arabs and Egyptians, and which is formed of cotton. The difficulty that Baron Percy found in burning these moxas without the aid of a blow-pipe, induced him to substitute the gun-match; and which he afterwards imitated by forming them from the stalk of the great sun-flower, steeped in a solution of nitre, and afterwards well dried. This, however, is objected to generally, because upon the *slow* combustion of the moxa the good effects are believed to depend.

Mr. Dunglison next proceeds to obviate some of the common objections made in this country to the use of this remedy, among the foremost of which is the pain said to attend its application. That this is not really so terrible as is represented, the authorities of Sir Wm. Temple, Van Swieten, and Pouteau, are adduced, all of whom tried it in their own persons; and as the first of these gentleman was not of the profession, nor in any way interested in the introduction or recommendation of this mode of cauterizing, his authority is of the more weight. In combating Mr. Cooper's objections to the use of moxa, we do not think Mr. Dunglison has been so successful; nor does M. Roux's experiments with it in this country prove more than that the pain is really not an objection to its employment. However, there can be no doubt that it has been neglected too much in this country; and, in truth, we believe that it has never yet had a fair trial.

We now come to the body of the work itself. We find that Baron Larrey forbids the application of the moxa in several parts of the body, of which the cranium, eye-lids, nose, ears, the larynx, sternum, the linea alba, and the course of the superficial tendons, are particularly mentioned; although other French writers do not agree in the justice of these restrictions; and, indeed, the Baron himself has broken through his own rules, as we shall afterwards find. He thinks that, together with the heat, a very active principle, which cottony substances furnish, is communicated to the parts. If superficial effects only are required, the cone may be permitted to burn down without the aid of the blow-pipe. To perform the operation, an instrument called a porte moxa is necessary; the handle of wood, with a circular metallic ring, in which the moxa is placed; and this ring is isolated from the skin by three small ebony balls. The cone is composed of a certain quantity of cotton wool, over which a piece of fine linen is rolled, and fastened at the side by a few stitches. This cone is about an inch long, and perhaps half an inch in circumference; but this may be varied according to circumstances. The pain, which during the commencement of the combustion is very slight, gradually increases, and is at length, he says, unquestionably very severe. The neighbouring parts are saved from the action of the heat by covering them with wet cloths; and, if suppuration be not desired, the liquor ammoniæ is poured upon the eschar, which dries it up, and it falls off in a few days in the form of a thin scale. He sometimes applies two cones at a time; but a few days should generally intervene between each application; for he thinks that many might produce not only too great a degree of pain, but such a suppuration as might be followed by hectic fever,—a position which we doubt, but which the Baron finds necessary to maintain, as it affords him a ground from preferring this his favourite remedy in caries of the spinal column. Moist weather is supposed not to be so favourable for this application; and cupping, either *mouchetées* or *scarifiées*, or even dry cupping, should be premised. Besides all this, the internal exhibition of remedies appropriated to each disease must be conjoined.

Here follow copious directions upon the subject of cupping, a subject which he thinks is better understood in France than in England; and he plumes himself very much upon the scarificator of his own invention, although evidently more painful and defective than the instrument which we employ, and of which he does not know the construction, since he fancies that we are not able to regulate the depth of our incisions. The difference between the *mouchetées* and the *scarifiées* is, in fact, no more than in degree; and the translator has made some very sensible observations upon this ebullition of French vanity.

We now come to the diseases for which the moxa has been employed, and we shall treat them in the order they occur, and as nearly in the terms of our author as possible, now and then adding such remarks as suggest themselves from the perusal of the cases.

*Vision.*—Defective action in the membranes of the globe of the eye, incipient cataract, and weakness or recent paralysis of the optic nerve, indicate the application of this remedy, which should be placed upon the principal ramifications of the fascial, frontal, and superior maxillary nerves. The moxa here may be used merely as an excitant, or suppuration may be permitted to take place. The cases requiring this are, he says, easily distinguishable; but he does not enter into particulars. He only illustrates this class of diseases by one case of partial amaurosis, and which has already appeared in the third volume of his "Campaigns."

We may dismiss the two next heads of *Smell* and *Taste*, the moxa having no effect upon diseases of these two senses. With regard to that of *Hearing*, when deafness is caused, he says, by the sedative action of cold, moxa is infinitely preferable to blistering. Of this he has witnessed numerous examples, but he inserts only one,—that of a trumpeter, who lost both his voice and sense of hearing in consequence of bathing in a state of perspiration. After cupping, and the application of thirteen moxas, this man was completely restored. In this soldier, it is added that both these affections had been considered as *feigned*.

In what M. Larrey calls paralytic affections of the muscular system, but which the detail of the cases proves to be *Tic Douloureux*, or chronic neuralgia, together with convulsive affections of the muscles, he advocates warmly the use of the moxa; but says that, in acute neuralgia, or in tetanus, it would not be equally indicated, because it increases the irritation. The cases which follow are not satisfactory: the first case was probably not tic douleureux; and there seem to have been a train of symptoms in the other two patients (females), and a mode of internal treatment adopted, which is not detailed, and therefore renders it impossible for us to judge what portion of the good effected must be ascribed to the moxa, and what to the internal remedies.

The subject of *Paralysis* occupies several pages. He notices the fact that sometimes, in these cases, the sensibility is destroyed, and the motion remains entire; and, on the contrary, the locomotive powers shall be entirely lost, and yet the sensations of the parts continue unimpaired. This remark is interesting, as connected with the experiments of M. Majendie, detailed in our last Number, and in consequence of which we shall not enter into the causes of these anomalies as explained by our author, and which the results of these experiments has rendered of no value whatever. In that description of case in which the powers of motion are lost, and those of sensation remain, the Baron urges the use of the moxa. In his first case, (that of M. P. counsellor of Paris,) however, we recognize a disease of the spine; for what else does the "jutting out" of the spinous processes of the tenth and eleventh dorsal vertebræ indicate? (p. 27,) and which, upon pressure, gave great pain. Moxas were applied in this situation to the amount of thirty-two, with intervals of some days, but the length of time occupied in the cure is not stated. The two following cases are loss of sensation in the fore-arm and hand, arising from the effects of gun-shot wounds: the use of the moxa restored both of these patients in some months.

"Hemiplegia of the face," says our author, p. 30, "has hitherto been considered as incurable by authors, because they durst not apply the moxa upon the face." To this we can only reply, that the partial paralysis of the face, from exposure to cold or other causes, in young subjects, is by no means considered as incurable on this side of the water, even without the aid of moxa; and we need only mention that, by repeated applications of this remedy, he succeeded in the cases of several soldiers, and in one young lady, whose disease, however, appears to us to have been very distinct in its nature, as it had existed from her infancy, and was attributed to a worm-fever. In this young lady, the cones were applied over the course of the trunk of the facial nerve, at its exit from the foramen stylo-mastoidean, thence following the direction of the principal branches of the nerve. The eschars were dried up by the fluid volatile alkali, in order to prevent deformity; and, after seventeen applications, the cure was nearly completed.

We pass over rather hastily the account of the success of our author in hemiplegia of the lower extremities and partial paraplegia, because there is nothing but an uniformity of practice and cure, excepting where these paraplegic cases are combined with incontinence of urine, in which M. Larrey does not expect the same fortunate result.

We here dismiss this long head, or division, from which we have not room to make any farther extract; and we think that a consideration of the various diseases jumbled together in this space will not afford a very favourable specimen of the arrangement of our author. With the exception of the moxa, no account is given of any of the curative methods employed in these cases; and the way in which many of them are dismissed, as "nearly well," or "almost cured," is far from satisfactory.

In *organic diseases of the Head*, including epilepsy, dropsy of the ventricles of the brain, chronic head-ach, &c. moxas are to be applied round the base of the cranium: higher upon the skull, their employment might, he thinks, produce unpleasant symptoms. Some cases, illustrating the practice, are given; among the most extraordinary of which is that of a young trumpeter, who, in consequence of a fall from his horse, was afflicted with epileptic fits for two years. The cranium had acquired in a short time such a size, that his uniform hat became five or six lines too narrow for his head: he was nearly paralytic, and all the sensitive functions were much impaired. After bleeding from the jugular, cupping, mustard-baths to the feet, and calomel internally, fifteen moxas were applied round the head. "The symptoms were gradually mitigated, so as to render the paroxysms milder and less frequent: at last they entirely disappeared, and the patient was perfectly cured before the end of the tenth month of his treatment;" and, when he was dismissed, behold! the hat, which had been five or six lines too small, was now as many lines too large, so that a reduction in the circumference of the head, of eight or ten lines, had taken place under the influence of the moxa!!!* Two or three cases follow, which our author denominates dropsy of the brain,—they all end well.

*Diseases of the Chest.*—When asthma is not hereditary, or produced by mal-conformation of the chest, moxa has been employed by M. Larrey with great success. Under this head, we have also some remarks upon neuralgic palpitations of the heart, proceeding from debility of that organ, and of the spinal marrow, all cureable by moxa; as are also catarrhal affections and chronic inflammation of the pleura.

Next in order follows the most appalling and startling section of the book, which is entitled *Phthisis Pulmonalis.*

At the risk of being thought tedious, we must quote the following passage:

"I might have been convinced, *a priori*, of the efficacy of this application, had I reflected upon the extraordinary success which I had obtained from its application in rachialgia and femoro coxalgia, which might be more properly termed spinal and articular phthisis, from which phthisis pulmonalis only differs in its seat. In fact, these two affections present the same phenomena, are owing to the same causes, and produce the same effects: besides, it frequently happens that diseased spine accompanies phthisis pulmonalis. In this latter disease, as in rachialgia, the moxa produces the discussion of the lympathic congestions or of the scrofulous tubercles, as well as of those abscesses which are symptomatic, when they are not too far developed. It cleanses the internal ulcers, stops the caries of the bones, and produces the adhesion and healing of the parietes of the abscess, or of the purulent cavities, established in the tissue of the lungs, or in any other part which may have become the seat of the phthitis. At last a cure, more or less complete, is obtained, according as the employment of this stimulating and revulsive agent has been more or less persevered in; an agent certainly but little used, but which experience has taught us to be most efficacious in those diseases. The internal remedies, more or less extolled by authors, (even acetate of lead, and, *a fortiori,* the prussic acid,) are generally hurtful, or at least quite useless. If, however, there should be any particular virus, it would be, first of all, necessary to remove that cause, and, when the phthisis alone remained, to attack it with the moxa."

After this, we really have not patience to do more than observe, that the two first cases narrated are not cases of phthisis. In both there was curvature of the spine,—one lateral, and the treatment of which occupied two years; that the third case is equally obscure, and one of the symptoms enumerated is "that the nails of the fingers were crooked." We can hardly persuade ourselves that M. Larrey has written this chapter in a serious

* Had not this man's head been shaved, or the hair cut close?

mood: he must surely have been disposed to try how far credulity could be carried; for it is scarcely possible that a man of his high reputation could have believed the contradictions and absurdities that abound throughout this section.

We must pass rapidly over one or two of the succeeding divisions, in order that we may be able to bestow more attention upon the disease which M. Larrey has denominated *Rachalgia*, or *maladie de Pott;* although, *en passant*, we agree with Mr. Dunglison in thinking that our author assumes too much in attributing the evacuation of hepatic abscesses by the intestines to the operation of the moxa; such a result being by no means unusual under any plan of treatment, and, indeed, sometimes under no treatment at all. A page or two is also devoted to *Rachitis*, and here we find, to our astonishment, that moxa is an undoubted cure: internal remedies are slightly mentioned, but only as auxiliaries.

*Rachialgia.*—This section begins thus: "The moxa is, above all, imperiously indicated in tabes dorsalis;" so that M. Larrey has got, for one and the same disease, no less than five names,—it is tabes dorsalis, rachialgia, vertebral disease, curvature of the spine, and maladie de Pott. He affirms that we are not commonly aware of its existence until it has arrived at its second or third stage; a position which we beg leave to deny altogether, although it is but too true that it is often suffered to arrive at its second stage before professional assistance is sought for. In this disease M. Larrey thinks the moxa "a sovereign remedy;" but, before he begins to treat upon it, he sets about what he calls reforming the phraseology, and substituting a name which may indicate the true character of the disease, which is "an inflammatory condition of the fibro-cartilaginous and osseous substance of the vertebral column, or of the boney substance of other parts of the trunk," and which he names according to its seat,—viz. Sacro-coxalgia, when it is situated in the sacro iliac symphyses; Sternalgia, when in the sternum; Costalgia, when upon the ribs or their cartilages; Scapulalgia, when in the scapula; and Femora-coxalgia, when seated in the coxo-femoral articulation. (p. 74.) We agree with the translator in condemning this barbarous jargon; besides, it conveys no idea of the disease, and the author departs from it immediately himself; for, in the very next page, he changes the first name, rachialgia, which he calls a *rheumatic* or scrofulous affection in some part of the vertebral column, which produces a latent or chronic inflammation in the fibro-cartilaginous and osseous tissues of the vertebræ: this, he says, is a *true phthisis*. We add the following specimen of the Baron's reasoning:—"This inflammation, far from augmenting, by turgescence, the volume of the parts, weakens their tissue, and appears to accelerate the work of absorption and of decomposition," &c. How refreshing is it, after this perusal, to turn to the sterling pages of POTT and BRODIE, and to mark the clearness and precision with which the history, causes, and progress of this malady are detailed, and the mode of treatment pointed out and illustrated. As usual, for the cure of this affection our author has nothing to recommend but cupping and the moxa; and it is curious that the cases he selects in this instance, as well as in most of his preceding ones, are any thing but fair specimens of the complaint which stands at the head of the section; and, what is no less so, he does not allude to the necessity of rest, or the advantages of the horizontal position, either in treating of the complaint or detailing the cures; although in one case he mentions it slightly and incidentally. We cannot afford space to detail these cases, or to comment upon them, though they afford much ground for so doing; but we may be allowed to say, that a cure, after two years, leaving the patient unable to bend his trunk forwards or sideways, (p. 87,) does not justify the Baron in boasting of the superiority of the moxa above the common cautery or issue, in the decided tone which he assumes upon this occasion. In the fifth case, again, the soldier, after about six months' treatment, "was in a way to convalescence," when, having been discharged, he returned home, "where the cure will, without doubt, have been completed." (p. 89.) Upon the seventh case we shall only remark, that in this instance there was an abscess in the left inguinal region, of the size of two fists, the bodies of the last dorsal vertebræ being the seat of the caries. This tumor M. Larrey evacuated by passing a seton through it, in the laudable design of causing the matter to be evacuated gradually. However, not being satisfied with the very marked reduction of this abscess, and fearing, from the thinness of its parietes, that it would burst spontaneously, he plunged *a red-hot knife into it;* and this is not the only instance in which he performed this neat operation. After this, he says, the patient was as well as *could be expected;* but, owing to an unfortunate indulgence in spirituous liquors, he died, after a month of anguish. Dissection showed what is usually termed a lumbar abscess of great extent, and a caries of the bodies of the third and fourth lumbar vertebræ.

We must pass over the reasoning suggested by this dissection, as well as a very long note upon syphilitic affections of bone, in no way connected with the subject before us; and, with regard to the remaining cases of this section, we shall only remark that the last is not a case of diseased spine, but a dislocation of one of the vertebræ, by a fall from a great height. Even this is put to rights by the moxa, after a treatment extended to a year and a half; at the end of which period the patient entreated to have amputation of the leg performed, the leg having been terribly fractured at the time of the fall, and so miserably united as to form an obstacle to his walking.

The next division is entitled on *Sacro-coxalgia*, and it is the last upon which we shall make any comment, since what our author calls the Femoro-coxalgia, known among us by the name of Disease of the Hip-joint, has been so ably illustrated in this country by the labours of Ford, Crowther, Baynton, Brodie, and others, that, with the exception of the substitution of the moxa for the seton, or caustic issue, we find nothing that deserves our notice. To return, then, to the sacro-coxalgia. M. Larrey believes that rheumatism may attack the sacro-iliac symphysis, so as to produce, in young persons, a gradual disunion of the two bones; though it is generally the result, he admits, of a mechanical cause. It also sometimes happens in very young women, in consequence of the birth of children of a disproportionate size. "The diagnosis of this complaint is difficult; the local pains, however, augmented by immediate pressure, and the manifest tumefaction in the sacro-iliac region, are sufficient to satisfy us of its existence." (p. 110.) The mode of cure is the usual one, the application of the moxa, but it must not be applied upon those portions of the skin which immediately cover the bones: the space, therefore, must be chosen which corresponds to the diseased symphysis.

The same affection, continues our author, sometimes also attacks the sternum, ribs, and scapula. "In all these cases, where abscesses form as a consequence of carious bone, and where they burst spontaneously before the caries of the bone is stopped by the means which I have made known, [i. e. cupping and moxa,] it is constantly mortal; but, if early use is made of the moxa, so as to arrest the progress of the caries, the operation pointed out for these abscesses, [that is, plunging a red-hot knife into them,] is attended with fortunate results." This concludes our author's account of a disease, of which he appears to have met with numerous examples. Caries of any bone in the body may certainly occur in consequence of constitutional disease or external injury; but the particular complaint which is described under the name of sacro-coxalgia is certainly, if not a novelty, a disease of very rare occurrence among us; and we are not aware that our author's explanation of the phenomena of the disease has ever been verified by dissection.

In conclusion, we cannot but express the astonishment with which we perused the greater part of this volume, and which we consider very unworthy of the high reputation the Baron has hitherto enjoyed. We fear that he is rather out of his element in these "piping times of peace," and that prompt decision and great manual dexterity in the field of battle are the distinguishing qualities of this excellent and veteran operator.

